# ADP-ribosylation-resistant rifabutin analogs show improved bactericidal activity against drug-tolerant *M. abscessus* in caseum surrogate

**DOI:** 10.1128/aac.00381-23

**Published:** 2023-07-26

**Authors:** Min Xie, Uday S. Ganapathy, Tian Lan, Paulina Osiecki, Jansy P. Sarathy, Véronique Dartois, Courtney C. Aldrich, Thomas Dick

**Affiliations:** 1 Center for Discovery and Innovation, Hackensack Meridian Health, Nutley, New Jersey, USA; 2 Department of Medicinal Chemistry, College of Pharmacy, University of Minnesota, Minneapolis, Minnesota, USA; 3 Department of Medical Sciences, Hackensack Meridian School of Medicine, Nutley, New Jersey, USA; 4 Department of Microbiology and Immunology, Georgetown University, Washington, District of Columbia, USA; Vanderbilt University Medical Center, Nashville, Tennessee, USA

**Keywords:** non-tuberculous mycobacteria, NTM, drug tolerance, persistence, caseum, rifamycins, ADP-ribosylase

## Abstract

Necrotic lesions and cavities filled with caseum are a hallmark of mycobacterial pulmonary disease. Bronchocavitary *Mycobacterium abscessus* disease is associated with poor treatment outcomes. In caseum surrogate, *M. abscessus* entered an extended stationary phase showing tolerance to killing by most current antibiotics, suggesting that caseum persisters contribute to the poor performance of available treatments. Novel ADP-ribosylation-resistant rifabutin analogs exhibited bactericidal activity against these *M. abscessus* persisters at concentrations achievable by rifamycins in caseum.

## INTRODUCTION

Treatment of lung disease caused by the opportunistic pathogen *Mycobacterium abscessus* delivers unsatisfactory cure rates despite the long-term use of multidrug regimens with an oral macrolide, clarithromycin, or azithromycin, as the backbone. First-line anti-tuberculosis (anti-TB) drugs are not used clinically due to intrinsic resistance (1). These include rifampicin and rifabutin, which *M. abscessus* inactivates by ADP ribosylation (2 - 4). Like TB, *M. abscessus* disease can be associated with necrotic lesions that contain extracellular bacilli, which can persist throughout antibiotic treatment (5). Necrotic lesions are filled with caseum, an acellular material that largely results from the necrosis of infected foamy macrophages (6). *Ex vivo* characterization of *Mycobacterium tuberculosis* in caseum derived from rabbit cavities showed that the bacteria are non-growing, retain viability for extended periods of time, and, importantly, show increased tolerance to killing by many TB drugs, with rifamycins retaining relevant bactericidal activity (7). Novel TB drugs that contribute to the eradication of this drug-tolerant bacterial subpopulation are expected to reduce treatment duration, as rifampicin did when introduced into the first-line regimen (8). To eliminate the reliance on *M. tuberculosis*-infected animals to generate caseous material for profiling discovery compounds, a caseum surrogate was developed using foamy macrophages grown in culture (9). The cell culture-derived surrogate recapitulates the lipid composition of native, animal-derived caseum, induces non-replicating survival of *M. tuberculosis,* and triggers physiologic and metabolic adaptations that lead to the drug-tolerant phenotype of *M. tuberculosis* found in animal-derived caseum (9). Here, we employed the caseum surrogate assay developed for *M. tuberculosis* to examine (i) the growth and survival of *M. abscessus*, (ii) the activity of clinically used antibiotics, and (iii) the bactericidal activity of novel rifabutin analogs that overcome intrinsic *M. abscessus* resistance through chemical modifications that block intrabacterial ADP ribosylation (10).

### *M. abscessus* survives and adopts an apparent non-growing state in surrogate caseum

**Fig 1:**
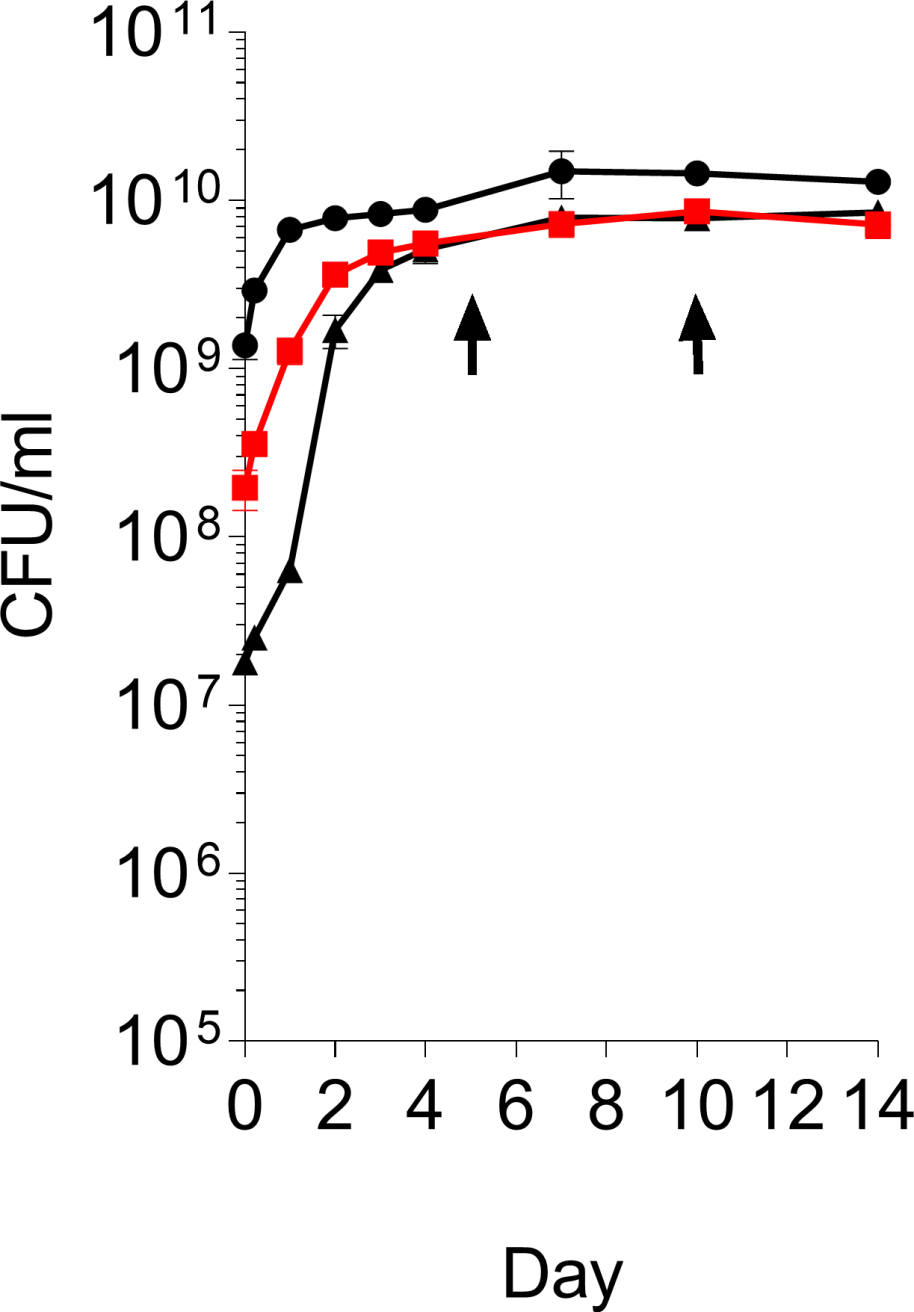
Growth of *M. abscessus* Bamboo in caseum surrogate. The surrogate matrix was generated as described previously from cultured THP-1 cells (ATCC TIB-202) (9). *M. abscessus* exponential cultures grown in Middlebrook 7H9 broth (Sigma Aldrich) (OD_600_ 0.6–0.9) were spun down and resuspended in water to an OD_600_ of 7, 0.7, and 0.07. As described for the *M. tuberculosis* caseum surrogate assay (9), the bacterial suspensions (at three different dilutions resulting in ~10^9^, 10^8^, and 10^7^ starting CFU/mL) were added to the caseum surrogate in the ratio 2:1 (vol/wt), briefly homogenized with 1.4-mm zirconia beads, divided evenly into nine 1.5-mL microcentrifuge tubes, and incubated as standing cultures at 37°C. At the indicated time points, tubes were removed and used for CFU enumeration by plating on Middlebrook 7H11 agar (Sigma Aldrich). Separate tubes were used at each time point. To determine the kill curves shown in Fig. 2, cultures with a starting CFU/mL of 10^8^ were used (resulting in the red growth curve). Arrows indicate the time points when drugs were added and the end of the treatment. The experiment was repeated three times independently, yielding similar results. A representative example is shown. Dots and error bars represent means and standard deviations of three technical replicates, respectively.

To determine whether *M. abscessus* is capable of surviving in caseum-like medium, a surrogate matrix was prepared from THP-1-derived macrophages exposed to stearic acid to induce the foamy phenotype as described (9) and inoculated with exponential phase *M. abscessus* subsp. *abscessus* Bamboo grown in Middlebrook 7H9 and resuspended in water. The Bamboo strain is a macrolide-sensitive, genome-sequenced clinical isolate (11). Serial sampling of inoculated caseum surrogate incubated at 37°C as standing culture (i.e., with limited oxygen supply, thus mimicking the situation encountered in caseous lesions and cavities) (9) for CFU enumeration on Middlebrook 7H11 agar showed that the cultures entered a stationary phase after an initial growth phase. CFU remained constant for up to 14 days, the duration of the experiment (Fig. 1). Thus, *M. abscessus* survives in caseum surrogate in an apparent non-growing state. To confirm that this also holds true in native caseum, the experiment was repeated using cavity caseum explanted from a rabbit infected with *M. tuberculosis*. Frozen rabbit caseum collected through independent studies (9) was used. The growth curves of *M. abscessus* in rabbit-derived caseum were similar (Fig. S1). It is to note that native caseum contains tubercle bacilli (up to 10^8^ CFU/g), introducing a potential confounding variable (7). Because of this, and with the caseum surrogate reproducing growth and survival of the bacterium shown in native caseum, subsequent experiments were carried out in caseum surrogate.

### Stationary phase *M. abscessus* grown in caseum surrogate is tolerant to killing by current antibiotics

Before measuring the drug tolerance of *M. abscessus* in caseum surrogate, we first determined the MIC and standard bactericidal activity of clinically used drugs against *M. abscessus* Bamboo cultures growing in Middlebrook 7H9 broth. MICs were derived from growth inhibition dose-response curves (Fig. S2) with OD_600_ as readout as described (12 - 15). The MICs, defined as concentrations that inhibit 90% of growth compared to untreated controls, are shown in Table 1. Bactericidal activity of drugs in growing *broth* cultures was measured by determining minimum bactericidal concentration 90 (bMBC_90_), defined as the minimum concentration that reduces the number of viable bacteria (measured by CFU enumeration on Middlebrook 7H11 agar with 0.4% charcoal) after 5 days of treatment by at least 90% compared to the starting inoculum. The broth-derived bMBC_90_ values are shown in Table 1 and were deduced from concentration kill curves (Fig. S3) generated as described (15).

**TABLE 1:** Broth MIC, broth MBC_90_ (bMBC_90_), and caseum surrogate MBC_90_ (cMBC_90_) of clinically used antibiotics and of novel ADP-ribosylation-resistant rifabutin analogs against *M. abscessus* Bamboo

Drug class	Drug	MIC (µM)	bMBC_90_ (µM)	cMBC_90_ (µM)	cMBC_90_/bMBC_90_
Macrolide	Clarithromycin	0.4	2	128	64
Aminoglycoside	Amikacin	25	40	12	0.3
β-Lactam (carbapenem)	Imipenem	10	18	42	2.3
β-Lactam (cephalosporin)	Cefoxitin	29	45	35	0.78
Glycylcycline	Tigecycline	12.5	8	8	1
Oxazolidinone	Linezolid	25	32	512	16
Diarylquinoline	Bedaquiline	0.4	2^*a*^	67^*a*^	33.5
Riminophenazine	Clofazimine	12.5	>128	>128	NA^*b*^
Fluoroquinolone	Moxifloxacin	6	2	8	4
Rifamycin	Rifabutin	3	5	72	16
Rifamycin	Rifabutin-5a	0.07	0.30	8	26.7
Rifamycin	Rifabutin-5m	0.02	0.16	2	12.5
Rifamycin	Rifabutin-5n	0.03	0.13	0.65	5

^*a*^ Assay extended to 10 days (instead of 5 days used for all other drugs) due to slow onset of bactericidal activity (16, 17).

^*b*^ NA: not applicable as not active.

**Fig 2:**
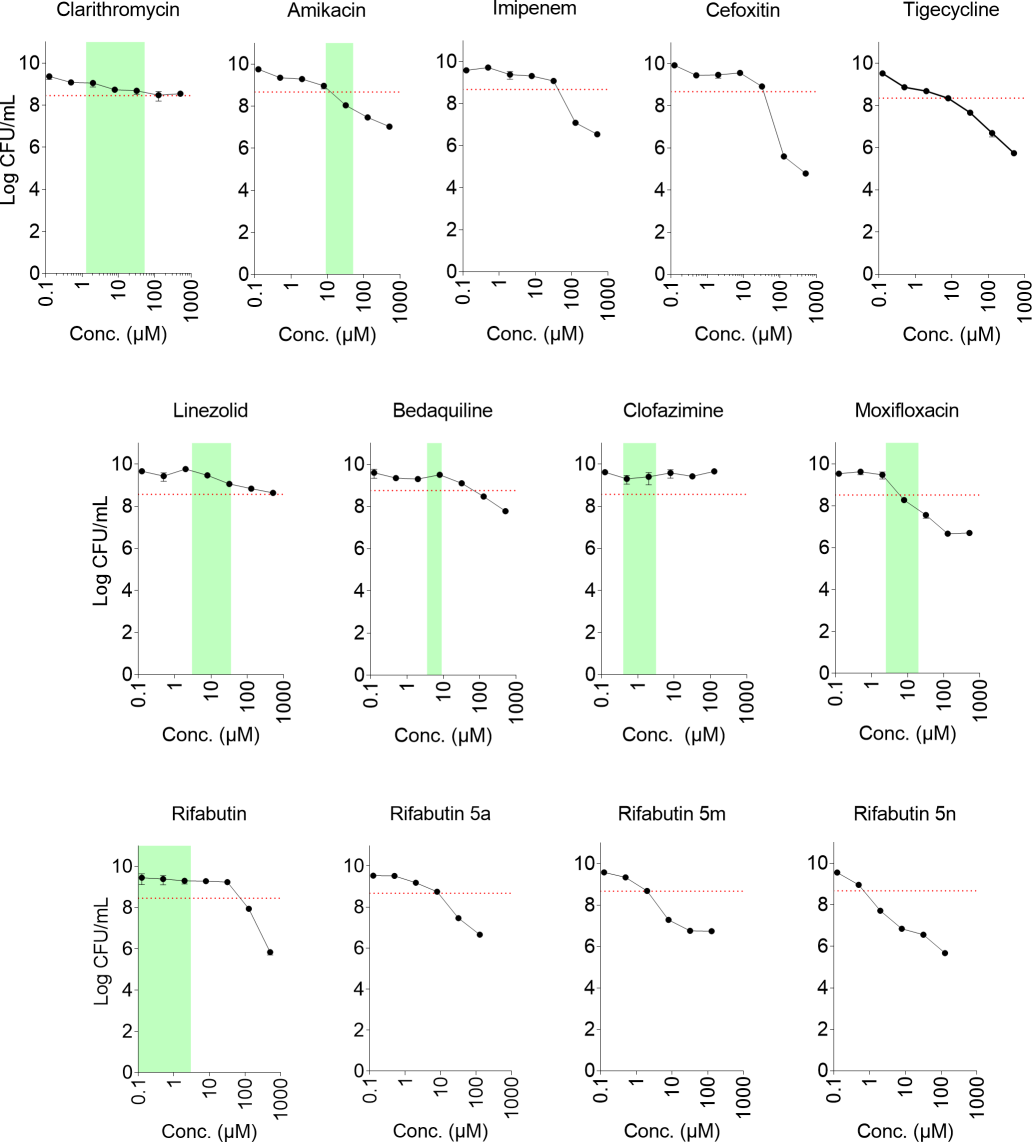
Dose-response kill curves against *M. abscessus* Bamboo in caseum surrogate. *M. abscessus* cultures were set up as described in the legend of Fig. 1. Bacterial cell suspensions were added to yield a starting CFU of 10^8^/mL (Fig. 1, red growth curve). At day 5, after the cultures entered stationary phase (Fig. 1, first arrow), 50 µL mixtures (cultures in caseum surrogate) were exposed to drugs (1 µL in DMSO, see Fig. S2 legend for drug sources) in the range of 0.125–512 µM (128 µM for clofazimine and rifabutin analogs 5a, 5m, 5n) for 5 days (or 10 days for bedaquiline as described in Table 1), after which CFU was enumerated. Addition of 2% of the vehicle DMSO did not affect viable counts. Green areas indicate drug concentration windows achieved in caseum *in vivo* [Table S1 (18 - 21) and Fig. S4]. The experiment was repeated twice independently, yielding similar results. A representative example is shown. Dots and error bars represent means and standard deviations of three technical replicates, respectively. Red dotted lines indicate the cutoff for 1 log reduction in CFU compared to 10-day drug-free control culture. The cMBC_90_ values shown in Table 1 are the drug concentrations that reduce CFU by 90% relative to the CFU of drug-free controls at day 10. Since the cultures were in the stationary phase on day 5 when drug treatment started, the CFUs of the drug-free cultures at day 10 were similar to the CFUs of the drug-free cultures at day 5 (Fig. 1).

To measure the bactericidal activity of standard-of-care antibiotics against non-growing *M. abscessus* adapted to caseum surrogate, bacteria were grown to stationary phase, then treated for 5 days, after which CFU were enumerated by plating on Middlebrook 7H11 agar supplemented with 0.4% charcoal. The concentration kill curves are shown in Fig. 2, and the derived *caseum* surrogate cMBC_90_s [defined as the minimum concentration that reduces the number of viable bacteria after 5 days of treatment by at least 90% compared to the (constant) drug-free stationary phase CFU] are shown in Table 1. Most drugs exerted reduced bactericidal activity against *M. abscessus* grown in caseum surrogate compared to broth-grown cultures, showing that *M. abscessus* becomes phenotypically drug resistant in a caseum-like environment. The quantitative impact of the caseum lifestyle on the bactericidal activity (cMBC_90_/bMBC_90_) was drug dependent and ranged from ~1 to 64 (Table 1). Importantly, the MBC_90_ of the key anti-*M*. *abscessus* macrolide clarithromycin increased 64-fold from 2 µM in broth to 128 µM in caseum. Another striking example of a drug losing bactericidal activity against non-growing “caseum-*M. abscessus*” was the F-ATP-synthase inhibitor bedaquiline showing a 34-fold shift of MBC_90_ from 2 to 67 µM. On the other hand, the aminoglycoside amikacin, the two β-lactams imipenem and cefoxitin, the glycylcycline tigecycline, and the fluoroquinolone moxifloxacin showed similar cMBC_90_ compared to bMBC_90_ (Table 1). Overall, the bactericidal activity of current antibiotics against caseum-adapted *M. abscessus* (Fig. 2) compared to broth-grown *M. abscessus* (Fig. S3) is variable but mostly reduced.

### Minimal bactericidal concentration in caseum surrogate (cMBC_90_) for most clinically used drugs is higher than concentrations achieved in necrotic lesions *in vivo*

Next, we asked whether the measured cMBC_90_s are achievable in necrotic lesions during therapy. Maximum and minimum achievable drug concentrations (*C*_max_, *C*_min_) (Table S1) were derived from previous pharmacokinetic studies on drug penetration into necrotic lesions [(18 - 21), Fig. S4]. Figure 2 shows that most drugs (clarithromycin, linezolid, bedaquiline, clofazimine, and rifabutin) fail to achieve concentrations that exert significant bactericidal activity against caseum-adapted *M. abscessus* (i.e., *C*_max_ < cMBC_90_) with the notable exception of amikacin and moxifloxacin. This suggests that most current anti-*M*. *abscessus* drugs are unable to significantly reduce the bacterial burden in necrotic lung lesions and cavities during treatment.

### ADP-ribosylation-resistant rifabutin analogs show improved bactericidal activity against *M. abscessus* in surrogate caseum

*M. abscessus* is intrinsically resistant to rifamycins. The RNA polymerase inhibitor rifabutin shows modest activity against *M. abscessus* (Bamboo MIC = 3 µM, Table 1 and Fig. S2) in broth cultures compared to its nanomolar activity against *M. tuberculosis* (7, 22, 23). Rifabutin’s bMBC_90_ against replicating *M. abscessus* Bamboo was 5 µM and shifted to 72 µM in the caseum surrogate assay (Table 1; Fig. S3; Fig. 2), far above the *C*_max_ (3 µM) achieved in necrotic lesions (Table S1; Fig. S4). Studies of the mechanism underlying intrinsic rifamycin resistance revealed that *M. abscessus* harbors an ADP-ribosylase that covalently modifies rifamycins and prevents engagement of their target (2 - 4). To restore the clinical utility of rifamycins for treating *M. abscessus* lung disease, we recently blocked the bacterial metabolism of rifabutin by chemical modification while retaining on-target and whole-cell activity (10). This delivered the potent leads 25-*O*-desacetyl-25-*O*-benzoylrifabutin (rifabutin-5a), 25-*O*-desacetyl-25-*O*-nicotinoylrifabutin (rifabutin-5m), and 25-*O*-desacetyl-25-*O*-pyrimidine-5-carbonylrifabutin (rifabutin-5n) (10) with MICs against *M. abscessus* Bamboo of 20 to 70 nM, approximately 40- to 150-fold more potent than rifabutin (Table 1; Fig. S2). We selected the three rifabutin analogs 5a, 5m, and 5n for bactericidal profiling because they showed similar *in vitro* activities but differed in their physicochemical properties (10). To determine whether the improved growth inhibitory activity translates into improved bactericidal activity against replicating and non-replicating *M. abscessus*, concentration kill experiments in broth and caseum surrogate were carried out (Fig. S3; Fig. 2). The lower MIC of rifabutin-5a, -5m, and -5n was associated with 15- to 40-fold lower MBC_90_ in broth, and 9- to 110-fold lower MBC_90_ against non-replicating persisters in caseum surrogate (Table 1). Interestingly, the bactericidal activity of the rifabutin analogs in caseum surrogate was proportional to their free fraction measured in plasma: 0.03%, 2.1%, and 4.7% for rifabutin-5a, -5m, and -5n, respectively (10), suggesting that non-specific binding to macromolecules affects their potency in this complex matrix since they exhibit similar growth inhibitory and bactericidal activity in broth (Table 1). Based on the superior mouse plasma PK profile of the three rifamycin analogs compared to rifabutin at the human equivalent dose or 10 mg/kg (Fig. S4A), and the favorable penetration of rifamycins from plasma to caseum at steady state in the rabbit model of active TB (Fig. S4B), it is estimated that rifabutin-5n may achieve cMBC_90_ in caseum throughout the dosing interval, as is the case for rifampicin in the treatment of TB, a feature critical to its remarkable efficacy (24). Collectively, our observations raise the possibility of ADP-ribosylation-resistant rifabutin analogs reducing bacterial burden in patients with cavitary disease.

### Conclusion

We show that *M. abscessus* adopts a non-growing, drug-tolerant phenotype in a surrogate, cell culture-derived caseum. For most anti-*M*. *abscessus* drugs, including the therapeutically critical macrolides, the concentrations reached in necrotic lesions *in vivo* are markedly below the concentrations required to exert significant bactericidal activity against caseum-adapted *M. abscessus*. These results identify *M. abscessus* residing in necrotic lesions as one of the possible causes for the prolonged and ineffective therapy. The caseum surrogate assay reported here may be a useful tool for screening discovery compounds against drug-tolerant *M. abscessus* and informing the development of more effective treatment regimens. Furthermore, the model can be used to understand the basis of drug tolerance in *M. abscessus* using transcriptomic analyses. To this end, we have applied the assay to show that ADP-ribosylation-resistant rifabutin analogs exert encouraging bactericidal activity against this form of non-growing *M. abscessus*, suggesting their potential to shorten treatment duration and reduce relapse rates.

## SUPPLEMENTAL MATERIAL

Supplemental Table S1, Fig. S1 to S4, references aac.00381-23-s0001.docx Table S1: PK; Fig. S1: growth curve; Fig. S2: MIC curves; Fig. S3: kill curves; Fig. S4: caseum PK; supplemental references.

ASM does not own the copyrights to Supplemental Material that may be linked to, or accessed through, an article. The authors have granted ASM a non-exclusive, world-wide license to publish the Supplemental Material files. Please contact the corresponding author directly for reuse.
