# Supplementary material for: ADP-ribosylation-resistant rifabutin analogs show improved bactericidal activity against drug-tolerant M. abscessus in caseum surrogate
Source: Antimicrob Agents Chemother. 2023 Jul 26;67(9):e00381-23. doi: 10.1128/aac.00381-23 (PMC10508146; doi:10.1128/aac.00381-23)
Supplement: Supplemental Table S1, Fig. S1 to S4, references — Table S1: PK; Fig. S1: growth curve; Fig. S2: MIC curves; Fig. S3: kill curves; Fig. S4: caseum PK; supplemental references. [file aac.00381-23-s0001.docx]

**Supplemental materials**

ADP-ribosylation resistant rifabutin analogs show improved bactericidal activity against drug tolerant *M. abscessus* in caseum surrogate

Min Xie,^a^ Uday S. Ganapathy,^a^ Tian Lan,^b^ Paulina Osiecki,^a^ Jansy P. Sarathy,^a^ Véronique Dartois,^a, c^ Courtney C. Aldrich,^b#^ Thomas Dick^a, c, d#^

^a^ Center for Discovery and Innovation, Hackensack Meridian Health, Nutley, New Jersey, USA

^b^ Department of Medicinal Chemistry, College of Pharmacy, University of Minnesota, Minneapolis, Minnesota, USA

^c^ Department of Medical Sciences, Hackensack Meridian School of Medicine, Nutley, New Jersey, USA

^d^ Department of Microbiology and Immunology, Georgetown University, Washington, DC, USA

Running Title: Rifamycin activity against drug tolerant *M. abscessus*

Keywords: Non-tuberculous mycobacteria, NTM, drug tolerance, persistence, caseum, rifamycins, ADP-ribosylase

^#^Address correspondence to Courtney Aldrich: [aldri015@umn.edu](mailto:aldri015@umn.edu), Thomas Dick: [thomas.dick.cdi@gmail.com](mailto:thomas.dick.cdi@gmail.com)

**Supplemental Table 1:** Peak and trough concentrations of standard of care drugs in cavity caseum

| Drug | MW | C_max_ caseum (ng/mL) | C_max_ caseum (μM) | C_min_ caseum (ng/mL) | C_min_ caseum (μM) | rabbit dose (mg/kg) | clinical dose (mg) | reference |
| --- | --- | --- | --- | --- | --- | --- | --- | --- |
| Clarithromycin | 748 | 40,000 | 53 | 1000 | 1.3 | 200 | 500 | (1) |
| Amikacin | 585.6 | 30,000 | 51 | 5,000 | 9 | 25 | 1,000 | (2) |
| Cefoxitin | 427.45 | No data | | | | | | |
| Imipenem | 317.36 |  |  |  |  |  |  |  |
| Tigecycline | 585.65 |  |  |  |  |  |  |  |
| Bedaquiline | 555.5 | 5,000 | 9.0 | 2000 | 3.6 | 125 | 400 QD loading | (3) |
| Moxifloxacin | 401.4 | 8,000 | 19.9 | 1000 | 2.5 | 40 | 400.0 | (4) |
| Clofazimine | 473.4 | 1,500 | 3.2 | 200 | 0.4 | na | 150 | (4) |
| Linezolid | 337.3 | 12,000 | 35.6 | 1000 | 3.0 | 90 | 1200 QD | (4) |
| Rifabutin | 847 | 2,500 | 3.0 | 100 | 0.1 | 15 | 300.0 | Fig. S4 |

**Supplemental figures**

**
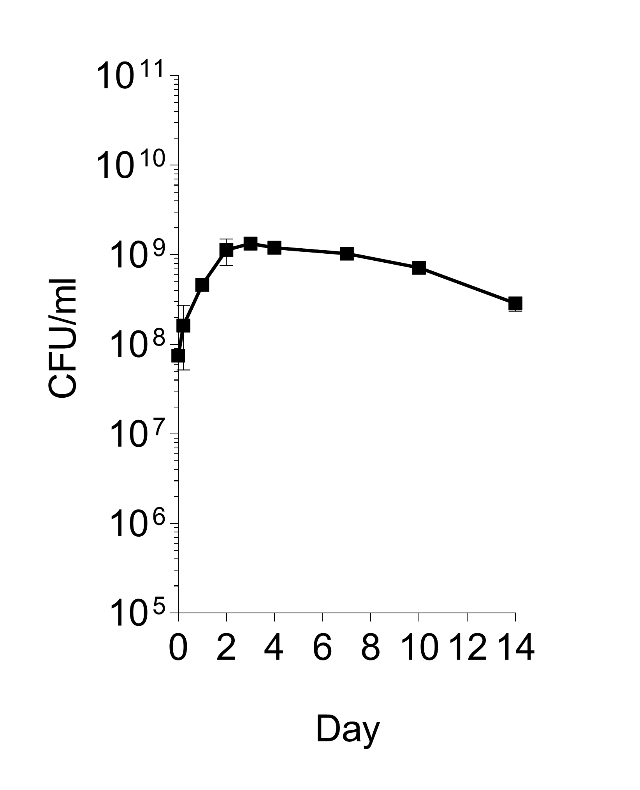
**

**Fig. S1.** Growth of *M. abscessus* Bamboo in caseum obtained from the cavities of rabbits infected with *M. tuberculosis* as described (5). *M. abscessus* cultures grown in 7H9 to an OD_600_ of 0.6 to 0.9 were spun down, resuspended in water to an OD_600_ of 0.7 and added to rabbit caseum in the ratio 2:1 (v/w) to achieve a starting inoculum of 10^8^ CFU/mL as described for the *M. tuberculosis* assay (5). The suspension was briefly homogenized with 1.4 mm zirconia beads, divided evenly into nine 1.5 mL microcentrifuge tubes, and incubated without shaking at 37 ºC. At the indicated time points, tubes were removed and used for CFU enumeration by plating on Middlebrook 7H11 agar. Separate tubes were used for each time point to avoid disturbing the culture upon repeated sampling. The experiment was repeated three times independently, yielding similar results. Means and standard deviations of three technical replicates of a representative example are shown.

**Fig. S2.** Growth inhibition dose response curves of *M. abscessus* Bamboo grown in 7H9 broth. MIC curves were generated as described using a 96-well broth dilution method with OD_600_ as readout (6-9). Exponentially growing *M. abscessus* cultures (OD_600_ = 0.5) were diluted to a starting OD_600_ of 0.05 and grown for three days at 37ºC. Dose response curves with means and standard deviations from two technical replicates are shown. Table 1 shows the corresponding MIC (defined as 90% growth inhibition compared to untreated control) of the various drugs derived from these dose response curves. The MIC values are similar to previously reported values (6-8, 10-14). Amikacin, clarithromycin, clofazimine, imipenem, rifabutin and tigecycline were purchased from Sigma Aldrich, moxifloxacin and linezolid from Sequoia Research Products, and cefoxitin and bedaquiline from MedChemExpress. The rifabutin analogs were synthesized as described (15).

**Fig. S3.** Dose response kill curves for *M. abscessus* Bamboo grown in Middlebrook 7H9 broth. Kill curves were generated as described using a 96-well format and enumeration of CFU on 7H11 agar after 5 days of treatment (9). Experimental cultures were set up from exponentially growing *M. abscessus* precultures as described in the legend of Fig. S2 for the generation of growth inhibition dose response curves. The deduced MBC_90_ values (minimum drug concentration resulting in a 90% reduction of CFU compared to the initial inoculum) are shown in Table 1. The experiment was repeated at least twice independently, yielding similar results. A representative example is shown. Dots and error bars represent means and standard deviations of three technical replicates, respectively. Red dots indicate the limit of CFU detection. Black dotted lines indicate the initial inoculum and red dotted lines indicate a 1 log CFU reduction compared to the initial inoculum. Green areas indicate drug concentration windows achieved in caseum *in vivo* ((1-4) and Fig. S4).

**Fig. S4**. Estimates of concentrations achieved in caseum versus cMBC_90_ for the novel rifabutin analogs. Animal studies were approved by the institutional animal care and use committee (IACUC) of Hackensack Meridian Health. **(A)** Plasma concentration time profiles of rifabutin (RBT) and the novel analogs in mice following a single oral dose of 10 mg/kg (the human equivalent dose for RBT), showing the superior exposure of RBT-5a, RBT-5m and RBT-5n compared to RBT. CD-1 mice were dosed as described in (15), n=3 for RBT, RBT-5m and RBT-5n; n = 6 for RBT-5a. Standard oral pharmacokinetic parameters are shown in the table. PPB: plasma protein binding; C_max_: peak plasma concentrations; AUC_[0-24]_: area under the concentration-time curve from 0 to 24h post dose; *f*AUC: free (unbound) AUC corrected for PPB. **(B)** Steady state plasma concentration time profiles of rifabutin (RBT) and mean concentrations in inner and outer cavity caseum following 7 daily doses of 15 mg/kg (the human equivalent dose) to New Zealand White Rabbits (n = 6). Drug penetration studies in rabbit lesions and cavities were carried out as described (1). The outer caseum encompasses a narrow band that directly subtends the cavity wall and cellular rim of closed lesions; the inner caseum is defined as the center of the necrotic core of cavities and closed necrotic lesions. The four dotted lines show the cMBC_90_ of RBT, 5a, 5m and 5n as indicated. Applying the extent of RBT penetration from plasma to caseum to the RBT analogs suggests that RBT-5n may achieve bactericidal concentrations against non-replicating caseum persisters in patients’ necrotic lesions and cavities.

**Supplemental references**

1. Kaya F, Ernest JP, LoMauro K, Gengenbacher M, Madani A, Aragaw WW, Zimmerman MD, Sarathy JP, Alvarez N, Daudelin I, Wang H, Lanni F, Weiner DM, Via LE, Barry CE, 3rd, Olivier KN, Dick T, Podell BK, Savic RM, Dartois V. 2022. A Rabbit Model to Study Antibiotic Penetration at the Site of Infection for Nontuberculous Mycobacterial Lung Disease: Macrolide Case Study. Antimicrob Agents Chemother 66:e0221221.

2. Ernest JP, Sarathy J, Wang N, Kaya F, Zimmerman MD, Strydom N, Wang H, Xie M, Gengenbacher M, Via LE, Barry CE, 3rd, Carter CL, Savic RM, Dartois V. 2021. Lesion Penetration and Activity Limit the Utility of Second-Line Injectable Agents in Pulmonary Tuberculosis. Antimicrob Agents Chemother 65:e0050621.

3. Irwin SM, Prideaux B, Lyon ER, Zimmerman MD, Brooks EJ, Schrupp CA, Chen C, Reichlen MJ, Asay BC, Voskuil MI, Nuermberger EL, Andries K, Lyons MA, Dartois V, Lenaerts AJ. 2016. Bedaquiline and Pyrazinamide Treatment Responses Are Affected by Pulmonary Lesion Heterogeneity in Mycobacterium tuberculosis Infected C3HeB/FeJ Mice. ACS Infect Dis 2:251-267.

4. Strydom N, Gupta SV, Fox WS, Via LE, Bang H, Lee M, Eum S, Shim T, Barry CE, 3rd, Zimmerman M, Dartois V, Savic RM. 2019. Tuberculosis drugs' distribution and emergence of resistance in patient's lung lesions: A mechanistic model and tool for regimen and dose optimization. PLoS Med 16:e1002773.

5. Sarathy JP, Xie M, Jones RM, Chang A, Osiecki P, Weiner D, Tsao WS, Dougher M, Blanc L, Fotouhi N, Via LE, Barry CE, 3rd, De Vlaminck I, Sherman DR, Dartois VA. 2023. A Novel Tool to Identify Bactericidal Compounds against Vulnerable Targets in Drug-Tolerant M. tuberculosis found in Caseum. mBio 14:e0059823.

6. Sarathy JP, Ganapathy US, Zimmerman MD, Dartois V, Gengenbacher M, Dick T. 2020. TBAJ-876, a 3,5-Dialkoxypyridine Analogue of Bedaquiline, Is Active against Mycobacterium abscessus. Antimicrob Agents Chemother 64:e02404-19.

7. Ganapathy US, Del Rio RG, Cacho-Izquierdo M, Ortega F, Lelievre J, Barros-Aguirre D, Aragaw WW, Zimmerman MD, Lindman M, Dartois V, Gengenbacher M, Dick T. 2021. A Mycobacterium tuberculosis NBTI DNA Gyrase Inhibitor Is Active against Mycobacterium abscessus. Antimicrob Agents Chemother 65:e0151421.

8. Ganapathy US, Del Rio RG, Cacho-Izquierdo M, Ortega F, Lelievre J, Barros-Aguirre D, Lindman M, Dartois V, Gengenbacher M, Dick T. 2021. A Leucyl-tRNA Synthetase Inhibitor with Broad-Spectrum Anti-Mycobacterial Activity. Antimicrob Agents Chemother 65:e02420-20.

9. Negatu DA, Beuchel A, Madani A, Alvarez N, Chen C, Aragaw WW, Zimmerman MD, Laleu B, Gengenbacher M, Dartois V, Imming P, Dick T. 2021. Piperidine-4-Carboxamides Target DNA Gyrase in Mycobacterium abscessus. Antimicrob Agents Chemother 65:e0067621.

10. Aziz DB, Low JL, Wu ML, Gengenbacher M, Teo JWP, Dartois V, Dick T. 2017. Rifabutin Is Active against Mycobacterium abscessus Complex. Antimicrob Agents Chemother 61:e00155-17.

11. Ganapathy US, Lan T, Krastel P, Lindman M, Zimmerman MD, Ho H, Sarathy JP, Evans JC, Dartois V, Aldrich CC, Dick T. 2021. Blocking Bacterial Naphthohydroquinone Oxidation and ADP-Ribosylation Improves Activity of Rifamycins against Mycobacterium abscessus. Antimicrob Agents Chemother 65:e0097821.

12. Madani A, Negatu DA, El Marrouni A, Miller RR, Boyce CW, Murgolo N, Bungard CJ, Zimmerman MD, Dartois V, Gengenbacher M, Olsen DB, Dick T. 2022. Activity of Tricyclic Pyrrolopyrimidine Gyrase B Inhibitor against Mycobacterium abscessus. Antimicrob Agents Chemother 66:e0066922.

13. Negatu DA, Zimmerman MD, Dartois V, Dick T. 2022. Strongly Bactericidal All-Oral beta-Lactam Combinations for the Treatment of Mycobacterium abscessus Lung Disease. Antimicrob Agents Chemother 66:e0079022.

14. Yam YK, Alvarez N, Go ML, Dick T. 2020. Extreme Drug Tolerance of Mycobacterium abscessus "Persisters". Front Microbiol 11:359.

15. Lan T, Ganapathy US, Sharma S, Ahn YM, Zimmerman M, Molodtsov V, Hegde P, Gengenbacher M, Ebright RH, Dartois V, Freundlich JS, Dick T, Aldrich CC. 2022. Redesign of Rifamycin Antibiotics to Overcome ADP-Ribosylation-Mediated Resistance. Angew Chem Int Ed Engl 61:e202211498.
